# Mapping *Lupinus polyphyllus* density and distribution along road verges using unmanned aerial vehicle (UAV)–based remote sensing

**DOI:** 10.1002/aps3.70080

**Published:** 2026-09-09

**Authors:** Elin L. Blomqvist, Jan‐Olov Andersson, R. Lutz Eckstein, Jan Haas

**Affiliations:** ^1^ Department of Environmental and Life Sciences Biology, Karlstad University 651 88 Karlstad Sweden; ^2^ Department of Environmental and Life Sciences Geomatics, Karlstad University 651 88 Karlstad Sweden

**Keywords:** invasive species, *Lupinus polyphyllus*, random forest, remote sensing, road verges, species distribution, unmanned aerial vehicle (UAV)

## Abstract

**Premise:**

Road verges function as refuges for semi‐natural species, but they can also facilitate the spread of non‐native plants such as *Lupinus polyphyllus*. Unmanned aerial vehicle (UAV)–based remote sensing is a promising tool for mapping these species; however, its application in roadside contexts remains limited.

**Methods:**

We developed and evaluated a UAV‐based workflow to detect and map lupine presence and density along roads. Red‐green‐blue (RGB) imagery was processed into orthomosaics and classified using object‐based and pixel‐based approaches with support vector machines and random forest algorithms under multiple classification schemes.

**Results:**

The highest accuracy (88.8%) was achieved using pixel‐based random forest classification and a simplified scheme. Flowering purple lupine showed strong classification performance (F1 score = 96.3%), while early‐stage greenish individuals were harder to detect (74.5%). Spatial heatmap comparison revealed low overestimation (1.79%) and underestimation (1.47%).

**Discussion:**

Assuming optimal phenological conditions, UAV‐based mapping provides a scalable and cost‐efficient complement to ground surveys for the management of *L. polyphyllus* within infrastructure areas.

Invasive alien species (IAS) are a major cause of global biodiversity loss, as recognized by the Intergovernmental Science‐Policy Platform on Biodiversity and Ecosystem Services (IPBES) (Roy et al., 2023). These species often spread beyond their native range due to human activity, establish populations, and disrupt native ecosystems by altering competitive interactions (Mooney and Cleland, 2001; Simberloff et al., 2013). Biological invasions not only lead to biodiversity loss but also impose significant economic costs due to management and control efforts (Born et al., 2005).

*Lupinus polyphyllus* Lindl. (hereafter referred to as lupine) is an invasive plant species native to western North America. It is a summer‐green perennial with rosette leaves that may reach a canopy height of about 40–90 cm (Ludewig et al., 2022). Each plant bears one to several stems, each ending in a single inflorescence ca. 40(–60) cm long (Ludewig et al., 2022). Individuals may vary in flower colors, ranging from whitish to yellowish via pink to purplish blue, with the latter flower morph being dominant over other colors (Bragdø, 1956). This high spectral variation poses a challenge for efficient classification approaches and automatic detection. Lupine has spread widely across Europe, occupying various habitats such as forestry areas, clear‐cuts, open mountain grasslands, forest edges, and road verges (Eckstein et al., 2023). Road verges have gained attention as important refuges for biodiversity, especially as semi‐natural grasslands degrade (Roy et al., 2023). However, due to their connectivity within the landscape, road verges can also facilitate the spread of IAS such as lupine (Lázaro‐Lobo and Ervin, 2019), which has been shown to negatively impact biodiversity in these areas (Valtonen et al., 2006).

Early detection of IAS is critical for preventing further spread and reducing control costs (Mehta et al., 2007), and remote sensing has proven to be an invaluable tool for monitoring and mapping IAS (Kennedy et al., 2009; Müllerová et al., 2023; Saranya et al., 2024). One approach is to use satellite imagery, which provides broad‐scale coverage and has shown success in mapping IAS (Royimani et al., 2019). However, choosing and acquiring appropriate remotely sensed data can be challenging due to atmospheric effects and cloud cover (Zhu and Helmer, 2018), insufficient spatial resolution, and inadequate temporal resolution, which can make it difficult to capture short flowering phases (Rahimi and Jung, 2025). In recent years, higher‐resolution data sources, such as aerial imagery from unmanned aerial vehicles (UAVs), have gained popularity for mapping plant species as they provide a more flexible, cost‐effective, and higher‐resolution alternative to traditional satellite‐based methods (Sishodia et al., 2020). These systems enable detailed monitoring of small areas with fine‐scale temporal resolution, making them particularly useful for monitoring IAS on a local scale (Anderson and Gaston, 2013). UAVs allow researchers to tailor data collection to specific needs, optimizing both spatial and temporal resolution (Tóth, 2018; Sun et al., 2021).

Several studies have tested UAV‐based methods for mapping invasive plant species, using a range of sensors from red‐green‐blue (RGB) cameras to hyperspectral sensors to capture different aspects of plant development (Rahimi and Jung, 2025). These methods have been applied to a variety of invasive plants, including woody species like *Acacia longifolia* (Andrews) Willd., *Rosa rugosa* Thunb., and *Pinus* L. (De Sá et al., 2018; Gonçalves et al., 2022; Bergamo et al., 2023), as well as herbaceous plants like *Iris pseudacorus* L., *Impatiens glandulifera* Royle, *Heracleum mantegazzianum* Sommier & Levier, and *Fallopia japonica* (Houtt.) Ronse Decr. (Hill et al., 2016; Michez et al., 2016a). However, the accuracy of mapping has varied due to factors such as species characteristics, sensor technology, and environmental conditions. Because phenological characteristics affect detection success, species‐specific approaches are essential for improving mapping accuracy (Lourenço et al., 2021). In a study on *L. polyphyllus*, Wijesingha et al. (2020) showed that combining UAV‐based RGB and thermal imagery with object‐based image analysis (OBIA) and random forest classification enables accurate detection in semi‐natural grasslands. Their best classification model achieved an overall accuracy (OA) of 97.2%, with canopy height model, spectral shape index, and object geometry as key predictors. The classified maps closely matched manually digitized maps, with a maximum difference of ±5% in total lupine cover. The high OA demonstrates the strong potential of UAV technology for mapping lupine, but landscape context may affect classification model performance. However, unlike relatively homogeneous grasslands, roadsides present additional challenges such as greater landscape heterogeneity, spectral variations in road surfaces, and tree shadows, which can contribute to misclassifications (Mansourmoghaddam et al., 2022; Chen et al., 2023). Building on the approach by Wijesingha et al. (2020), considerations such as UAV flight altitude and sensor configuration can influence both data acquisition efficiency and the amount of pre‐processing required. Approaches relying solely on RGB imagery offer a simpler sensor setup compared to methods that incorporate thermal imagery and canopy height modeling. Previous studies have also shown that shadows can negatively affect UAV‐based detection of IAS (Müllerová et al., 2017; De Sá et al., 2018; Lopatin et al., 2019).

Currently, lupine populations in Sweden are inventoried in situ using a method developed by Helldin et al. (2024) within the TRIEKOL/TRIIAS project for the Swedish Transport Administration. The method involves assessing lupine coverage along the road network, with occurrences identified from cars, and is used to inform planning for lupine control efforts (Helldin et al., 2024). Lupine occurrences are classified into five categories based on coverage: dominant (>90% coverage), dense (50–90% coverage), sparse (<50% coverage), variable (where density varies within a stand, e.g., a dense core surrounded by sparser coverage), and patchy (where lupine patches are scattered along a longer stretch, making individual documentation impractical). A single occurrence class is used for occurrences with a density of less than 1 m^2^. Helldin et al. (2024) highlight the difficulty of accurately assessing coverage, especially for tall species like lupine, where varying leaf heights can lead to subjective assessments and observer bias. They stress the need for more efficient methods, particularly along major roads, where car‐based inventories are costly and require the use of accompanying safety vehicles. While UAV technology is suggested as a potential solution, it has yet to be tested, and the authors also note concerns regarding the accuracy of remote sensing for road verge inventories.

Hence, the aim of this study was to develop and evaluate a workflow for mapping the occurrence and density of lupine along roads using UAV technology. This approach could serve as a complement to or even replace traditional in situ inventory methods, with the potential to support long‐term monitoring and follow‐up control measures. By providing detailed spatial data on lupine distribution, the workflow could form a basis for assessing the effectiveness of management efforts and facilitate a scalable and cost‐efficient alternative to conventional mapping techniques.

## METHODS

### Study area

For this study, a 250‐m road segment in Värmland County, Sweden (59.458972°N, 13.139500°E), was selected (Figure 1). This road segment is representative of road verges with dominant stands of garden lupine along small gravel roads that are managed by private road associations. This type of road usually represents most of the length of regional road networks, especially in rural areas in Sweden (e.g., Helldin et al., 2022). The selected road segment runs through flat, open, agricultural ley farming fields embedded in a forest landscape, which is a typical landscape structure for much of western Sweden (Sporrong et al., 1995). Therefore, we consider the chosen site as being representative of road verges in large parts of western Sweden and probably even beyond.

**Figure 1:**
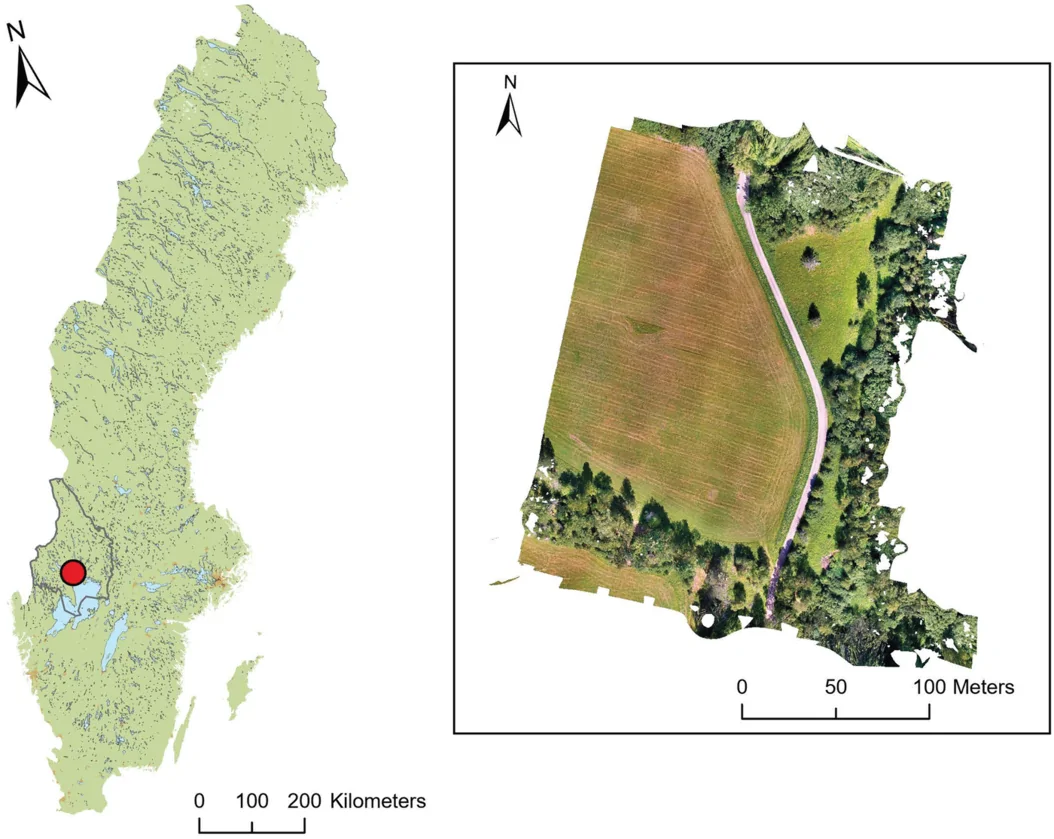
The location of the study area in Sweden, marked with a red dot within the boundaries of Värmland County, outlined in gray. Data source: © Lantmäteriet (left), used with permission. On the right, an orthomosaic of the study area is shown.

To develop a method capable of handling high spectral variation, we specifically selected a challenging site where diverse flower colors and phenological stages co‐occurred. Classification accuracy is affected along shady, forested roads due to increased shadowing and background heterogeneity. Shadows from trees further increase classification complexity; previous studies have highlighted how shadows influence spectral classification of vegetation (Müllerová et al., 2017; De Sá et al., 2018; Lopatin et al., 2019), making this an important factor to consider in a road verge context.

### UAV data

The study area was delineated in ArcGIS Pro v. 3.4.2 (Esri, Redlands, California, USA) by creating a shapefile polygon, which was then imported into DroneDeploy (San Francisco, California, USA) for flight planning and execution. Data acquisition was conducted on 11 July 2022, using a DJI Phantom 4 Pro (DJI, Shenzhen, China) equipped with an RGB camera (1″ CMOS sensor, 20 MP effective pixels) at an altitude of 35 m, which was empirically determined as the most suitable (altitudes from 10 to 120 m were tested). The flight was completed with an 80% front overlap and a 75% side overlap.

### Validation data

Validation data were collected manually on the orthomosaic through a point vector layer, sampling at the center of each lupine inflorescence across the entire study area. Points were only placed where lupine inflorescences could be confidently identified. The dataset was then categorized into three groups: purple lupine, pink lupine, and greenish lupine (the latter representing individuals in both the early flowering stage and the overflowered stage with immature seed pods, as these were found to exhibit similar spectral characteristics in a preliminary classification test). In total, 11,133 lupine inflorescences were manually annotated, comprising 8136 purple lupines, 1197 pink lupines, and 1800 greenish lupines. Validation points were also created for the other classes that were sufficiently represented in the landscape, with 1000 points each for vegetation, dry vegetation, shaded vegetation, road, shaded road, and 600 points for branches. Both UAV data collection and creation of the validation dataset were performed by the author with the most knowledge of the study site (E.L.B.). There are no other independent datasets available that can be used for validation purposes.

### Photogrammetric processing

The overall workflow of data acquisition, photogrammetric processing, classification, and spatial analyses is illustrated in Figure 2. The images were imported into Agisoft Metashape Professional (Agisoft, St. Petersburg, Russia), where an orthomosaic was created using the standard workflow for processing RGB images (Appendix S1). High accuracy was applied during photo alignment, followed by the construction of a dense point cloud and a digital elevation model. The resulting orthomosaic was georeferenced with a spatial resolution of 8.25 mm/pixel.

**Figure 2:**
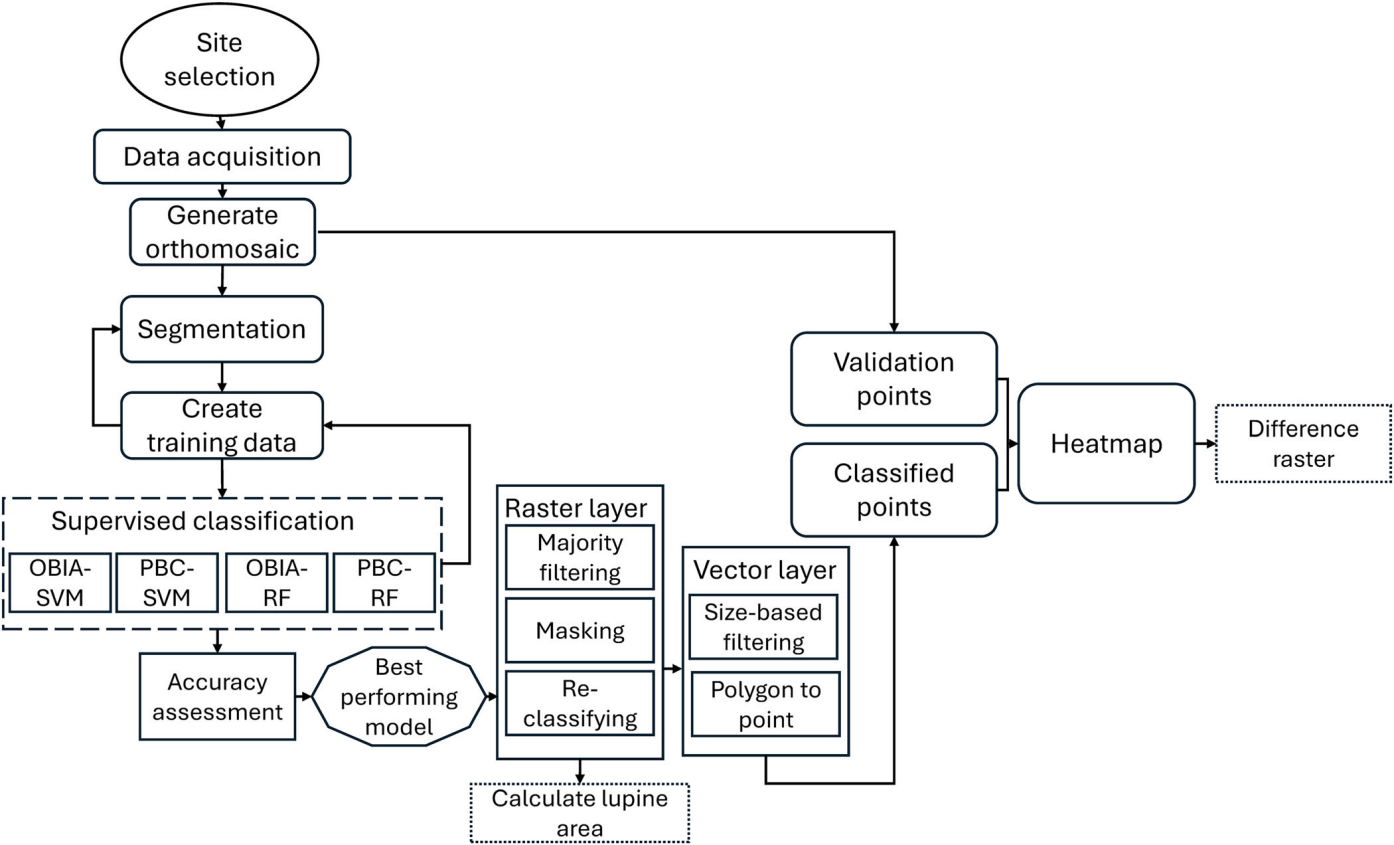
Flowchart for remote sensing data analysis. Double‐framed rectangles represent the lupine occurrence results derived from the classification. OBIA, object‐based image analysis; PBC, pixel‐based classification; RF, random forest; SVM, support vector machine.

### Image segmentation

Prior to segmentation, the orthomosaic was clipped to a relevant subsection containing the road area (see Appendix S2), which served as the region of interest. Image segmentation based on the mean shift approach (Comaniciu and Meer, 2002) was then performed to group pixels into objects or features that shared similar spectral and spatial characteristics (Chen et al., 2018). Multiple parameter values were tested, and the settings were chosen based on a visual comparison of the segmented images with the RGB imagery of the study area (Appendix S3). These segments were subsequently used to create the training data.

### Training data

To develop a robust classification model, we first identified key land cover classes within the orthomosaic, focusing on the dominant vegetation types and surface characteristics to ensure comprehensive representation in the training data. The initial classification scheme included nine information classes: purple lupine, pink lupine, greenish lupine, general vegetation, shaded vegetation, dry vegetation, road, shadowed road, and branches. During preliminary classification trials, however, we observed that including road‐related classes introduced confusion between classes, particularly affecting the distinction between purple and pink lupine. To address this, we tested an alternative classification scheme, removing the road and road shadow classes while maintaining the same training areas for the other categories. Careful selection of training samples is essential for accurate classification, as well‐chosen training areas capturing relevant and diverse features improve model performance while minimizing the influence of redundant or irrelevant data (Georganos et al., 2017). To ensure that representative segments were selected for each class, we used the Segment Picker tool in the Training Samples Manager in ArcGIS Pro on the segmented layer and validated the selection against the orthomosaic to ensure alignment with the intended class.

### Classification and accuracy assessment

Support vector machine (SVM) and random forest (RF) were selected as classifiers due to their proven effectiveness in remote sensing applications, particularly for classifying IAS with high accuracy (Sabat‐Tomala et al., 2020; Nininahazwe et al., 2023). RF is a non‐parametric ensemble learning method that constructs multiple decision trees from randomly selected training data and input variables. It is computationally efficient, robust against overfitting, and handles correlated variables and imbalanced data well (Breiman, 2001). In contrast, SVM is a supervised learning algorithm based on statistical learning theory, which identifies an optimal hyperplane to maximize class separation by using a subset of critical training samples known as support vectors (Vapnik, 1999; Huang et al., 2002).

Two supervised classification approaches were applied: pixel‐based classification (PBC) and OBIA. PBC relies solely on spectral information, treating each pixel independently (Tehrany et al., 2014), whereas OBIA segments the image into homogeneous objects, incorporating spatial characteristics such as shape, size, texture, and contextual relationships (Blaschke, 2010). OBIA has gained popularity for mapping IAS due to its improved classification accuracy (Pande‐Chhetri et al., 2017; Abeysinghe et al., 2019; Lourenço et al., 2021). For instance, Michez et al. (2016a) found that fine OBIA segmentation (scale = 10) provided the best results for classifying *Impatiens glandulifera* when using flowers as sample units. However, in some cases, PBC outperformed OBIA, particularly when segmentation was too generalized (Martínez Prentice et al., 2021; da Silva et al., 2023).

To assess the impact on classification performance of including road classes and branches, we tested three classification schemes: one with road classes and branches, one with road classes excluded, and one without both road classes and branches. Each scheme was applied using both OBIA and PBC, with SVM and RF as classifiers. This resulted in a total of 12 classification models:

With all classes: OBIA‐SVM, OBIA‐RF, PBC‐SVM, PBC‐RF

Without road classes (NoRC): OBIA‐SVM, OBIA‐RF, PBC‐SVM, PBC‐RF

Without road and branch classes (NoRBC): OBIA‐SVM, OBIA‐RF, PBC‐SVM, PBC‐RF

For both OBIA and PBC, training samples were selected using the Segment Picker tool, ensuring consistency in the training data across methods. The validation dataset was used to evaluate classification performance, and classification accuracy was assessed by extracting raster values corresponding to validation points using the Extract Multi Values to Points tool in ArcGIS Pro. These values were compared to the reference data, and confusion matrices were generated to calculate the following performance metrics: precision (Equation 1), recall (Equation 2), F1 score (Equation 3), and OA (Equation 4). In addition to these traditional accuracy measures, we also calculated macro‐average metrics to better assess the model's performance across all classes. Macro‐average computes the average of precision, recall, and F1 score for each class separately, treating all classes equally regardless of their frequency in the dataset (Grandini et al., 2020). This metric is particularly useful when dealing with imbalanced datasets, as it ensures that each class contributes equally to the overall performance evaluation. The model with the highest OA was selected for further analysis. To improve the quality of the classification results, the Majority Filter tool was applied. This step helped to smooth the output and reduce noise by eliminating isolated misclassified pixels:

(1) $$\mathrm{Precision}=\frac{\mathrm{TP}}{\mathrm{TP}+\mathrm{FP}}$$

(2) $$\mathrm{Recall}=\frac{\mathrm{TP}}{\mathrm{TP}+\mathrm{FN}}$$

(3) $$F1\;\mathrm{Score}=2\times \frac{\mathrm{Precision}\times \mathrm{Recall}}{\mathrm{Precision}+\mathrm{Recall}}$$

(4) $$\mathrm{OA}=\frac{\mathrm{TP}+\mathrm{TN}}{\mathrm{TP}+\mathrm{TN}+\mathrm{FP}+\mathrm{FN}}$$

where TP signifies true positive, FP false positive, TN true negative, and FN false negative.

Systematic misclassifications were identified after initial assessment of classification and accuracy. To mitigate these errors, a manual masking step was performed by creating polygon masks over areas where misclassifications frequently occurred. These included lupine‐free roads, which were often confused with lupine classes, and dense tree canopies and branches. Thereafter, ArcGIS Pro's Extract by Mask tool was used to refine the classification results. This tool was applied to exclude areas outside the manually created masks, ensuring that misclassified regions did not interfere with subsequent analyses. The masked areas were assigned “no data” values.

### Heatmap

Two heatmaps were generated: one based on manually created validation data and the other derived from the classified lupine occurrences. For the heatmap based on manual data, classified lupine inflorescences were first vectorized. To reduce overestimation caused by misclassification, one correctly classified lupine inflorescence was identified as the minimum size threshold. Polygons below that threshold were assumed to be false positives and were filtered out. Following polygon filtering, the remaining polygons were converted to point features by extracting their centroids. These points were then used as input for the Kernel Density tool to generate the heatmap. The population field in the tool was set to “none” so that each point was only counted once. This method was chosen because a single plant may have multiple inflorescences and plants often grow in dense clusters, making accurate plant counts difficult; this approach ensures that the density map reflects spatial patterns rather than exact flower numbers. The search radius was set to 1 m, which was chosen to estimate local lupine density consistent with Helldin et al. (2024). Output cell values represented density estimates.

A separate heatmap was generated for the validation dataset using the same Kernel Density parameters as used for the classified lupine data. Lupine density was visualized in six manual intervals: no occurrence, ≤1, ≤3, ≤6, ≤12, and ≥12 points/m^2^ in both maps. We chose manual intervals for comparative reasons, but other clustering methods such as the Jenks optimization method (Jenks, 1967) are viable alternatives. In order to compare the validation heatmap with the classified heatmap, a difference raster was generated between the maps to visualize underestimation and overestimation of lupine occurrences. An occurrence represents an individual lupine inflorescence rather than a plant, as a single plant may produce multiple inflorescences. Differences exceeding ±5 occurrences/m^2^ were considered overestimation or underestimation; this symmetric threshold was chosen to account for within‐plant flowering variability and minor smoothing effects from the Kernel Density analysis. Differences within ±5 occurrences/m^2^ were considered negligible for the spatial comparison. To quantify the overestimation and underestimation in the generated heatmap based on classified lupine occurrence, the areas corresponding to overestimated and underestimated pixels were calculated. The total area of these regions was derived by multiplying the pixel count by the area represented by each pixel (8.25 mm/pixel). The percentage of overestimation and underestimation was then calculated by dividing the respective areas by the total heatmap area.

### Lupine area

To estimate the classified area of lupine inflorescences within the study area, the raster generated from the best‐performing classification model (NoRBC PBC‐RF) was used. The three lupine classes were reclassified into a single lupine class, while all other classes were set to “no data.” The total lupine‐inflorescence area was then calculated by multiplying the number of pixels belonging to the lupine class in the masked raster by the pixel area (m^2^), based on image resolution.

## RESULTS

### Image segmentation

During segmentation, the parameters were carefully adjusted to avoid overly generalized segments while preventing excessively small segments, which would increase processing time. A high minimum segment size resulted in larger, more generalized segments that often included surrounding vegetation, leading to classification issues as lupine segments contained pixels from other classes. To mitigate this, a lower segment size threshold was necessary. High spectral detail was chosen as it best preserved the spectral differences between lupines and the surrounding vegetation. Higher spatial detail values produced smaller segments that closely followed object boundaries; however, further increasing spatial detail resulted in the algorithm creating segments that were perceived as unnecessarily small and unrepresentative of larger objects. The optimal balance was achieved with the selected parameters, which preserved sufficient object delineation while minimizing unnecessary fragmentation and processing time (Figure 3).

**Figure 3:**
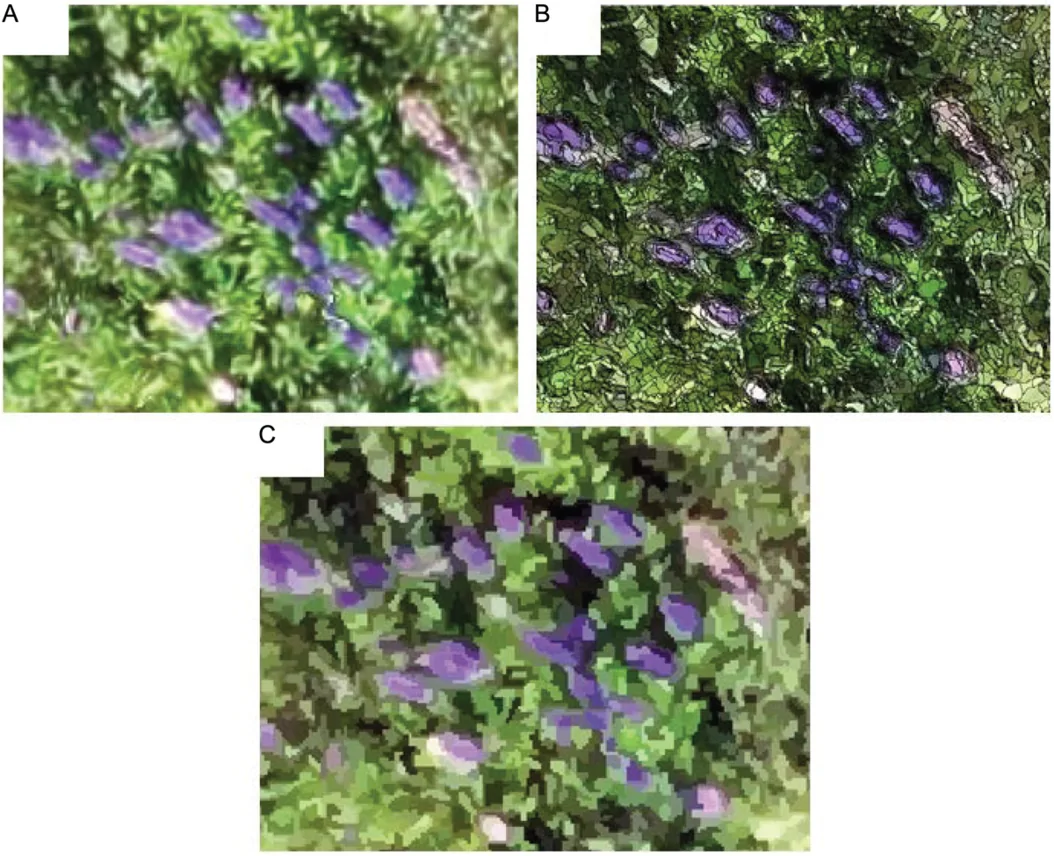
RGB image before segmentation (A), preview of segmentation (B), and final segments used for creating the training data (C).

### Classification results

We tested three different classification schemes: (1) using training data for all classes, (2) excluding road classes, and (3) removing both road classes and branches. When confusion matrices were created for the different classification models, we found that the model performance metrics improved as the training scheme was reduced (Table 1).

**Table 1:** Comparison of model performance for support vector machine (SVM) and random forest (RF) classifiers using object‐based image analysis (OBIA) and pixel‐based classification (PBC) approaches. Precision, recall, and F1 score are calculated using the macro‐average, and overall accuracy (OA) is reported as percentage. Results are shown for three classification schemes: all training classes included, road classes removed (NoRC), and both road and branch classes removed (NoRBC).

Model	Precision (%)	Recall (%)	F1 score (%)	OA (%)
All classes				
OBIA‐SVM	62.7	65.3	64.0	69.5
PBC‐SVM	70.8	76.5	73.5	75.5
OBIA‐RF	64.2	68.6	66.3	71.9
PBC‐RF	67.5	71.7	69.6	74.3
Road classes removed (NoRC)				
OBIA‐SVM	68.7	74.2	71.4	77.4
PBC‐SVM	75.0	77.7	76.3	83.7
OBIA‐RF	59.1	67.4	63.0	72.6
PBC‐RF	73.0	74.9	73.9	81.5
Road classes and branches removed (NoRBC)				
OBIA‐SVM	73.4	77.3	75.3	81.2
PBC‐SVM	81.6	84.7	83.1	88.3
OBIA‐RF	70.8	76.7	73.6	80.8
PBC‐RF	83.0	83.9	83.5	88.8

Both road classes and the branch class posed challenges for the classification process, making it difficult to distinguish them from lupine classes and generating noise in lupine occurrences (Figure 4). Spectral variations within these classes (introduced by shading) made it challenging to create representative training areas, as the training classes exhibited a high degree of internal variation. Higher accuracy was achieved when the road and branch classes were removed from the classification scheme (Appendices S4–S15). Pixel‐based classification achieved a higher OA (SVM = 88.3%, RF = 88.8%) compared to object‐based classification (SVM = 81.2%, RF = 80.8%). The best‐performing model was NoRBC PBC‐RF, which yielded the highest OA (Figure 5). Among the lupine classes, the highest accuracy was achieved for purple inflorescences, with an F1 score of 95.8%, followed by pink inflorescences (85.3%), while greenish lupines had the lowest F1 score among the lupine classes at 70.4% (Table 2). The primary source of false positives for greenish lupines was the purple lupine class. A possible explanation for this is that during seed pod development greenish lupines often retain flowers on the inflorescence, creating spectral overlap between the classes. As a result, the model may misclassify these individuals as purple lupines, especially when the color transition is gradual or when the flowers still exhibit a distinct purple hue. After applying the Majority Filter tool, accuracy improved slightly, with the F1 score for purple lupines increasing to 96.3%, for pink lupines to 87.9%, and for greenish lupines to 74.5% (Table 3). The F1 scores for vegetation classes ranged from 72.5% to 92.5%; the improvement in F1 scores suggests that the Majority Filter tool helped reduce noise and isolate misclassifications by smoothing small, fragmented misclassified areas.

**Figure 4:**
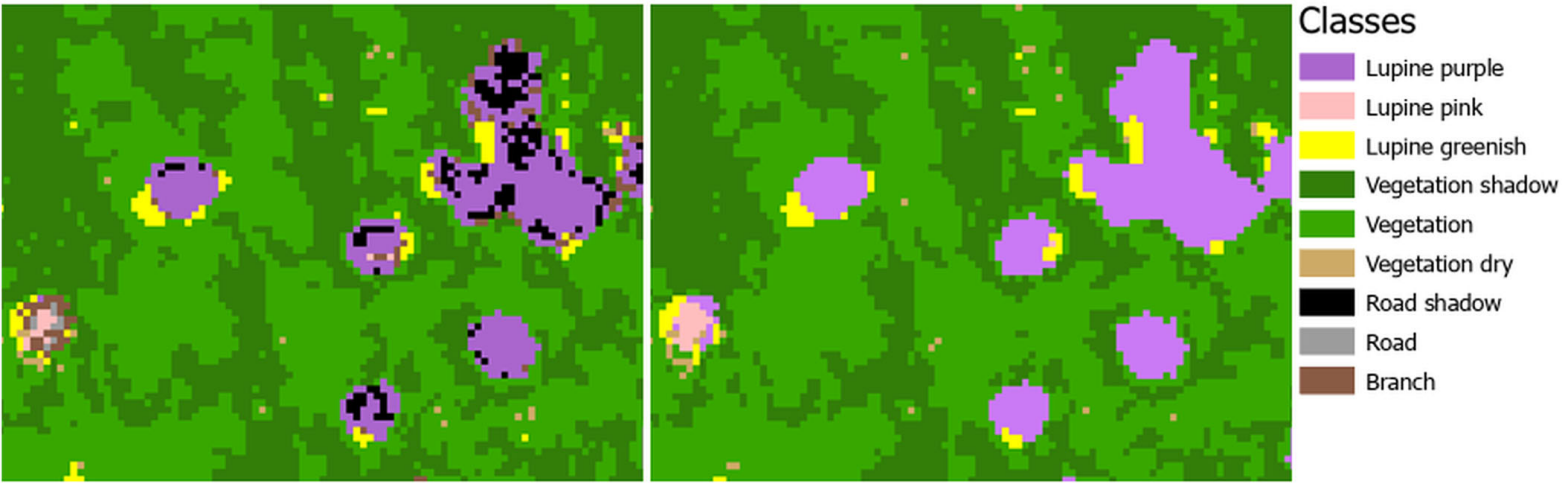
A snippet from the pixel‐based random forest (RF) classification map with all classes included (left) and the PBC‐RF without the road and branch classes (right). Road classes and the branch class introduce noise in the classified lupine areas.

**Figure 5:**
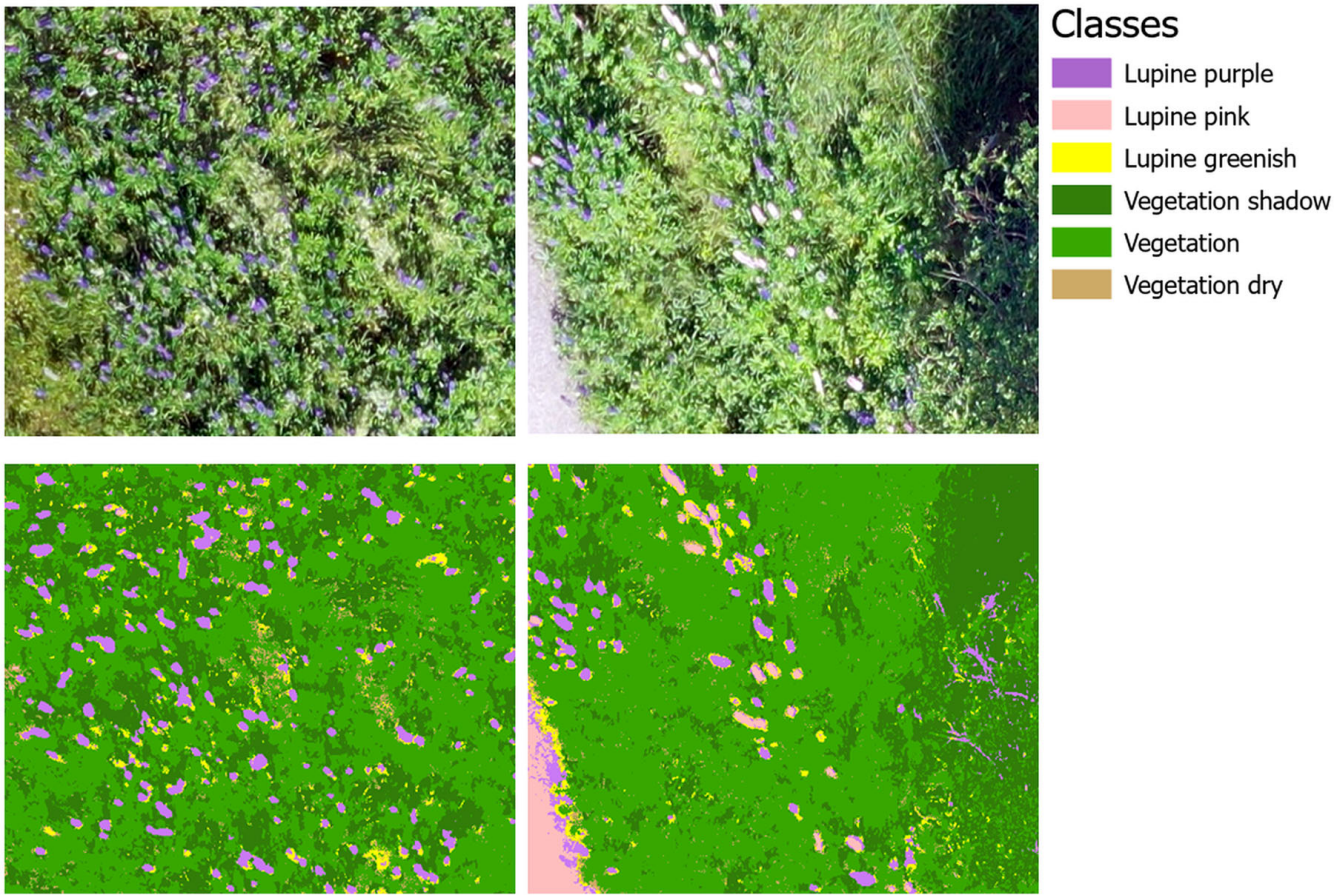
The top row shows the orthomosaic, while the bottom row shows the classified raster from the NoRBC PBC‐RF model. The columns represent the same subsets of the study area.

**Table 2:** Confusion matrix for the best‐performing model (NoRBC PBC‐RF).

Class	Reference data						Precision (%)	Recall (%)	F1 score (%)
	Purple lupine	Pink lupine	Greenish lupine	Veg. shadow	Veg.	Veg. dry			
Purple lupine	**7882**	57	117	21	95	4	95.2	96.4	95.8
Pink lupine	101	**963**	28	1	8	102	91.2	80	85.3
Greenish lupine	300	32	**1062**	6	239	161	87.2	59	70.4
Veg. shadow	0	0	0	**981**	19	0	86.4	98.1	91.9
Veg.	0	0	0	0	**999**	1	65.8	99.9	79.3
Veg. dry	0	4	11	126	159	**700**	72.3	70	71.1
Total land cover (%)	8.8	17.4	1.2	25.1	41.9	5.5			

**Table 3:** Confusion matrix for the best‐performing model (NoRBC PBC‐RF) after applying the Majority Filter tool.

Class	Reference data						Precision (%)	Recall (%)	F1 score (%)
	Purple lupine	Pink lupine	Greenish lupine	Veg. shadow	Veg.	Veg. dry			
Purple lupine	**7912**	27	106	16	111	4	95.8	96.8	96.3
Pink lupine	92	**981**	29	1	9	91	95.3	81.5	87.9
Greenish lupine	253	19	**1154**	6	225	143	88.8	64.1	74.5
Veg. shadow	0	0	0	**985**	15	0	87.2	98.5	92.5
Veg.	0	0	0	0	**1000**	0	65.7	100	79.3
Veg. dry	0	2	10	121	163	**704**	74.7	70.4	72.5
Total land cover (%)	8.7	17.5	1.1	25.3	42.7	4.7			

When training data for the road and branch classes were removed from the classification scheme, noise within actual lupine occurrences decreased. However, this also introduced a substantial overestimation of lupine presence in the area, as roads and branches were misclassified as purple and pink lupine (Appendix S16). Masking and excluding these areas significantly reduced the number of misclassified lupine pixels in the study area in a time‐efficient manner without compromising the model's overall ability to detect actual lupine occurrences. By masking these classes, we effectively reduced spectral misclassification between non‐vegetated surfaces, tree canopies, and lupine classes, preventing a systematic overestimation of lupine distribution.

### Heatmaps

Based on 11,179 validation points for lupine inflorescences and 11,198 classified lupine points (after size filtering of noise), two separate heatmaps were generated. The largest difference between the heatmaps, where the difference exceeds five occurrences/m^2^, is shown in red for underestimation of the classified raster compared to the validation raster and in yellow for overestimation of the classified raster compared to the validation raster (Figure 6). Overestimation of lupine occurrence in the classified raster was attributed to misclassifications, whereas underestimation occurred during raster‐to‐vector conversion, when polygons were created that represented multiple inflorescences within a single polygon (Appendix S17). This resulted in closely spaced inflorescences being represented as a single polygon, which, when converted to points, led to fewer points being generated than the actual number of lupine inflorescences. As a result, a single polygon represented multiple points, causing underestimation in areas with high lupine density. The overestimated areas had an interval range of −19.9 to −5 occurrences/m^2^, while underestimated areas had an interval range of 5 to 15.7 occurrences/m^2^. Overestimation accounted for 1.79% and underestimation for 1.47% of the total area in the classified heatmap.

**Figure 6:**
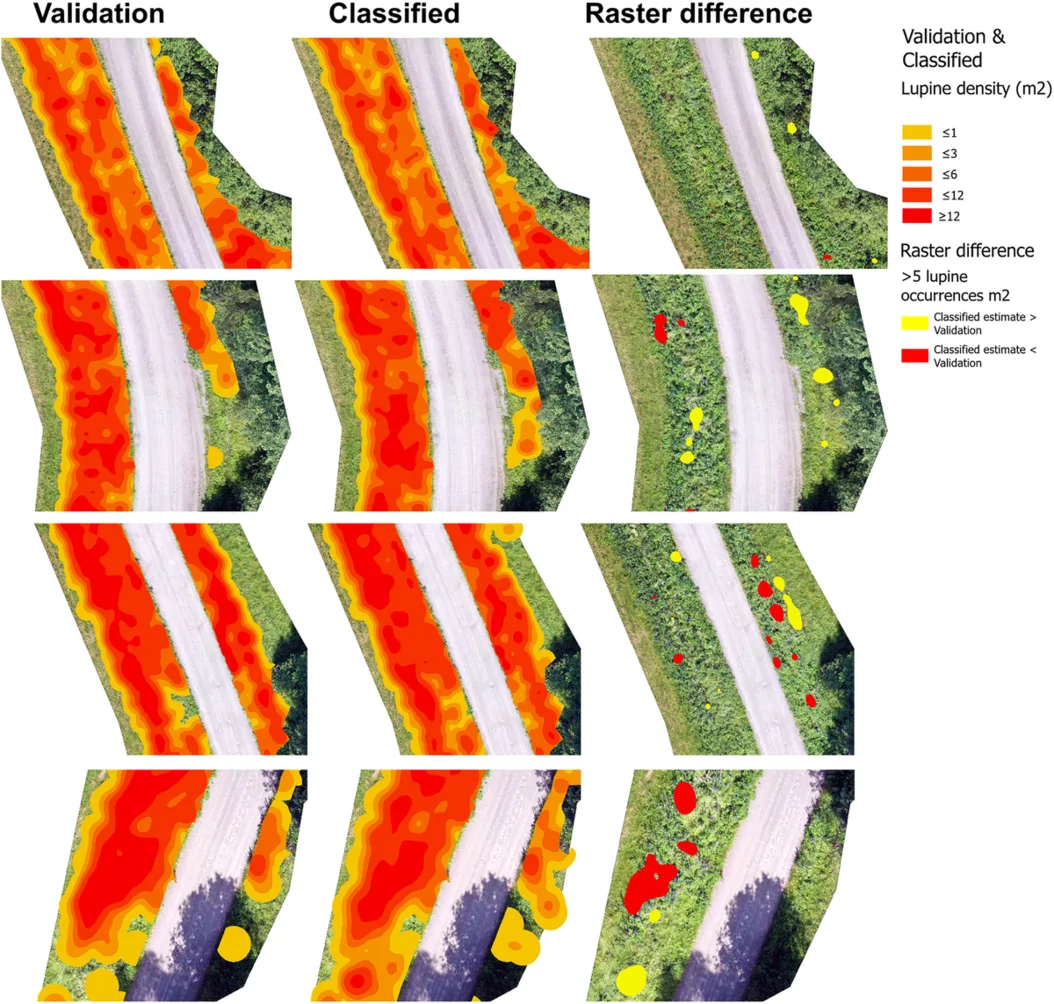
The rows display the same area from the study site, with the first column showing a heatmap generated from the validation data, the second column showing a heatmap generated from the classified lupine occurrence, and the third column displaying the difference between the two rasters. In the heatmaps, darker red shades represent higher lupine occurrence/m^2^. In the difference raster, red areas indicate where the classified raster underestimates lupine occurrence by more than five occurrences/m^2^ relative to the validation raster, while yellow areas indicate where the classified raster overestimates lupine occurrence by more than five occurrences/m^2^ relative to the validation raster.

### Lupine area based on pixels

The orthomosaic covered a total area of 2.25 ha, while the total classified lupine area (including all lupine classes) was 75.1 m^2^. To relate this to real‐world measurements, the diameter of a lupine inflorescence viewed from above is approximately 6 cm. Given the inflorescence's roughly circular shape, the area was estimated using the formula for the area of a circle. Multiplying this area by the total number of validation lupine points (11,179) resulted in an estimated total area covered by lupine inflorescences of 31.53 m^2^. A key limitation of this approach, however, is that lupine inflorescences were rarely captured in a perfectly top‐down view during data acquisition, meaning their actual surface area is likely larger. An alternative method based on the classified lupine points used to generate heatmaps (11,198 points) resulted in an estimated total lupine area of 31.65 m^2^.

## DISCUSSION

This study demonstrates that UAV‐based mapping can be an effective method to identify and quantify the occurrence of lupine along roadsides with high accuracy, assuming the environmental challenges are properly addressed. We achieved an OA of 88.8% using a pixel‐based random forest (PBC‐RF) classification approach in a complex environment. In comparison, Wijesingha et al. (2020) reported an OA of 97.2% using an OBIA‐RF model when mapping lupine in semi‐natural grasslands. This difference in accuracy may be partly attributed to differences in landscape context, which can influence the spectral and structural separability of classes. Semi‐natural grasslands typically exhibit a more homogeneous vegetation structure, dominated by herbaceous plants and grasses of similar height. In contrast, roadside environments are often affected by surrounding objects such as trees, fences, and other infrastructure, which contribute to greater spectral and structural variation within the imagery. As a result, optimizing segmentation parameters to capture object variation within a scene is challenging.

Landscape complexity could therefore be one of the reasons why the PBC approaches in our study outperform OBIA. Moreover, Wijesingha et al. (2020) employed a more advanced segmentation technique that optimizes parameters for each image region, likely leading to better segmentation outcomes and improved performance with OBIA, although it is difficult to measure segmentation quality and impact on classification accuracy. However, this segmentation requires additional data that was not used in our study. When no segmentation optimization technique is applied, OBIA may present challenges such as selecting an appropriate segmentation scale, preparing training samples, and assessing accuracy (Hao et al., 2021). A further limitation is that the OBIA segmentation process can lead to overly generalized class representations, which may cause small or isolated objects to be lost or misclassified (da Silva et al., 2023).

These factors could have contributed to the lower performance observed with OBIA in our study, highlighting the advantages of the PBC method in this case. Our approach complements the study of Wijesingha et al. (2020), showcasing effective lupine detection in complex landscapes. The proposed method relies solely on RGB data at a higher flight altitude (35 m vs. 20 m), rendering the approach both more efficient in terms of data collection and less costly as no additional equipment or advanced processing are needed. This is in line with the statement of Wijesingha et al. (2020) that, as temperature is not a significant lupine predictor, costs are reduced for sensors and platforms, while model complexity and computing time are decreased. It could be worthwhile to investigate whether including a canopy height model in complex heterogeneous landscapes would contribute to higher lupine detection rates.

As observed in previous studies mapping IAS (Müllerová et al., 2017; De Sá et al., 2018; Lopatin et al., 2019), shadowing from trees and other structures introduced significant challenges during classification and led to high misclassification rates. This is likely due to the difficulty in delineating representative training areas when a class exhibits high intra‐class spectral variability, which reduces model performance. Furthermore, these classes may share similar spectral characteristics with other land cover types, further complicating the classification process. The gradient of shadow variation is particularly difficult to capture accurately, and generating separate subclasses for this variation would be time‐consuming. Because the goal is to develop a classification workflow for lupine that is straightforward and easily applicable in practice, the method should not require advanced remote sensing knowledge or species‐specific expertise. Masking out areas where lupine does not grow or cannot be reliably detected, such as tree canopies and road surfaces, helped reduce the time spent on optimizing training data while still achieving high OA.

Among the lupine classes, the model performed best at identifying purple lupines (F1 score = 96.3%), while accuracy was lowest for greenish lupines (F1 score = 74.5%). The primary source of false positives for greenish lupines was confusion with purple lupines, which can be explained by the fact that the species simultaneously carries both flowers and seed pods during its development (Blomqvist et al., 2025a), resulting in a single inflorescence sometimes exhibiting both purple and green hues. This also occasionally caused difficulties in determining the correct class for validation data.

The higher accuracy in identifying purple lupines can be attributed to their distinct characteristics, which make them easier to differentiate from the surrounding vegetation. As highlighted by Bradley (2014), successful plant detection relies on clear distinguishing features. The bright color of the purple lupine flowers likely provided a strong contrast against the green background, in contrast to the greenish lupines, which were spectrally more similar to the surrounding vegetation. This underscores the need for clearly distinguishable features during both segmentation and classification. As shown by Wijesingha et al. (2020), hue was particularly effective for defining object boundaries in UAV imagery, facilitating accurate segmentation of individual plants. Given that segmentation quality directly affects the selection of training samples, this aligns with previous findings emphasizing the value of well‐defined and diverse training data for improving model performance (Georganos et al., 2017). In this context, the distinct coloration of purple lupines likely enhanced both segmentation and classification accuracy, while the spectral similarity between greenish lupines and the surrounding vegetation contributed to misclassifications.

While TRIEKOL/TRIIAS's field‐based inventory method is well suited for large‐scale mapping of lupine presence across the landscape, the UAV‐based approach used in this study offers advantages for population‐level analysis. By applying the same parameters and analytical methods as outlined in the workflow, future studies could compare changes in lupine populations over time, providing more consistent and comparable results. This could be particularly useful for assessing the effectiveness of control efforts. TRIEKOL/TRIIAS's method relies on subjective visual estimates of cover and density, which may introduce observer bias and reduce comparability over time or between observers (Helldin et al., 2024). In contrast, the PBC and density estimation applied here generate quantitative outputs that are less susceptible to personal interpretation, thus facilitating more accurate long‐term monitoring of population‐level dynamics.

Helldin et al. (2024) reported that during car‐based inventories the probability of detecting a single lupine plant was 65%, while dense stands were detected with 97% accuracy. About 73% of missed observations were individual plants, demonstrating that it is more challenging to identify small occurrences. Detecting these populations early is crucial for managing invasive species effectively, as it may make eradication feasible and help reduce control costs (Mehta et al., 2007). Our UAV‐based approach, with a high F1 score for the most common color variant of lupine, may contribute to better detection of individual inflorescences in both sparse and dense populations. Although the method employed in this study proved effective for mapping lupine, it is important to note that its direct application requires access to specific software. In this study, we used DroneDeploy for flight planning and data collection, Agisoft Metashape for photogrammetry and point cloud generation, and ArcGIS Pro for data processing and analysis. While these platforms are commonly used in remote sensing and UAV‐based mapping, there may be alternative software solutions that could offer similar performance, although these have not been tested in the current study.

Timing UAV data collection to coincide with the most distinct phenological stages between species is key to improving classification accuracy, as demonstrated by Michez et al. (2016b), who found that data collected during these periods yielded higher accuracy. This supports our findings, suggesting that capturing species during these stages can enhance classification performance and reduce the risk of misclassification due to variations in development. However, this approach also places significant demands on data collection, as it must be conducted at the right time each year and during the same developmental phase to ensure comparability of results. This challenge is further compounded by within‐population variation in phenological development (Blomqvist et al., 2025a), making it difficult to define an optimal period for data collection.

One solution to this challenge could be the inclusion of additional plant traits to expand the collection window. By integrating the distinct leaf shape and color plus plant height as classification features, the dependence on inflorescence could be mitigated. To achieve this, we suggest the use of a near‐infrared or red‐edge band or a hyperspectral sensor in addition to another segmentation strategy that increases the influence of spatial characteristics. Band rationing or using an index‐based approach could further make the detection approach more generic. Additionally, weather conditions such as wind and rain can impose further constraints, limiting the ability to collect data under ideal conditions. Although the timing of data collection places certain demands on planning, it also creates opportunities for more precise and effective monitoring. UAV‐based mapping provides detailed spatial information that can support targeted follow‐up of control efforts and help optimize resource use (Prior et al., 2018). This approach strengthens the foundation for evaluating management outcomes and improving strategic decision‐making in the control of lupine.

This study shows that a straightforward data‐efficient UAV‐based workflow can achieve high accuracy in mapping lupine in road verges. The approach does not require advanced remote sensing expertise, making it suitable for practical applications. The timing of data collection proved to be an important factor for obtaining high‐quality data, as the phenological stage of the species influences classification accuracy. The method also shows strong potential for supporting the follow‐up of control measures by providing spatially detailed and consistent data. The study has empirically determined effective parameters for lupine classification in complex environments based on RGB UAV imagery; it presents a straightforward approach that complements field‐based inventories and highlights both its potential and limitations. Low‐cost or open‐source alternatives such as QGIS and OpenDroneMap could be considered for increasing workflow efficiency and automation, thus reducing manual steps and fieldwork. Future studies could also focus on change detection methodological development, enabling more efficient long‐term monitoring of lupine.

## AUTHOR CONTRIBUTIONS

E.L.B., J.H., R.L.E., and J.O.A. contributed to the conceptualization of the study. Material preparation and data collection were performed by E.L.B. and J.O.A., who also handled data curation. Data analyses were carried out by E.L.B. with input from J.H. E.L.B. wrote the original draft of the manuscript, while J.H., R.L.E., and J.O.A. contributed to review and editing. Supervision of the research, including oversight and guidance throughout the study, was provided by J.H., R.L.E., and J.O.A. All authors approved the final version of the manuscript.

## Supporting information


**Appendix S1:** Workflow and parameters for generating the RGB orthomosaic in Agisoft Metashape Professional v. 2.2.0.
**Appendix S2:** Orthomosaic of the study area (left) with a clipped subsection (right) focusing on the road verges.
**Appendix S3:** Parameters used for image segmentation, including their descriptions and input data.
**Appendix S4:** All classes OBIA‐SVM.
**Appendix S5:** All classes OBIA‐RF.
**Appendix S6:** All classes PBC‐SVM.
**Appendix S7:** All classes PBC‐RF.
**Appendix S8:** NoRC OBIA‐SVM.
**Appendix S9:** NoRC OBIA‐RF.
**Appendix S10:** NoRC PBC‐SVM.
**Appendix S11:** NoRC PBC‐RF.
**Appendix S12:** NoRBC OBIA‐SVM.
**Appendix S13:** NoRBC OBIA‐RF.
**Appendix S14:** NoRBC PBC‐SVM.
**Appendix S15:** NoRBC PBC‐RF.
**Appendix S16:** Classified raster maps before (left) and after (right) masking of roads. The left image shows the initial classification, where the road is misclassified as pink and purple lupine. The right image illustrates the result after masking and excluding the road surface, reducing misclassification in the study area.
**Appendix S17:** Over‐ and underestimation of lupine occurrence. The red areas represent underestimation, while the yellow areas indicate overestimation in relation to the validation data. The points represent validation data for lupine, and the blue polygons show the classified lupine occurrence.

## Data Availability

Training data for this study are available at Zenodo (https://doi.org/10.5281/zenodo.17165184; Blomqvist et al., 2025b).
