# Supplementary material for: Mapping Lupinus polyphyllus density and distribution along road verges using unmanned aerial vehicle (UAV)–based remote sensing
Source: Appl Plant Sci. 2026 Sep 9;14(5):e70080. doi: 10.1002/aps3.70080 (PMC13559118; doi:10.1002/aps3.70080)
Supplement: Supplementary file 1 [file APS3-14-e70080-s001.docx]

**Appendix S1.** Workflow and parameters for generating the RGB orthomosaic in Agisoft Metashape Professional v. 2.2.0.

| Orthomosaic generation | |
| --- | --- |
| 1. Align photos | a. Reference preselection |
|  | b. High accuracy |
|  | c. Key point limit - 40,000 |
|  | d. Tie point limit - 4000 |
| 2. Build dense cloud settings | a. Quality - High |
|  | b. Depth filtering - Mild |
| 3. Build DEM | a. Type - Geographic |
|  | b. Interpolation - Enabled |
| 4. Build orthomosaic | a. Type - Geographic |
|  | b. Surface - DEM |
|  | c. Blending mode - Mosaic |
|  | d. Enable hole filling selected |

**Appendix S2.** Orthomosaic of the study area (left) with a clipped subsection (right) focusing on the road verges.

**Appendix S3.** Parameters used for image segmentation, including their descriptions and input data.

| Parameter | Description | Input data |
| --- | --- | --- |
| Input raster bands | Raster data used for segmentation | RGB orthomosaic   (3 bands) |
| Spectral detail | The degree of importance assigned to the spectral differences of features in the imagery. | 19 |
| Spatial detail | The degree of importance assigned to the spatial proximity between features in the imagery. | 16 |
| Minimum segment size in pixels | The minimum size of a segment, below which segments will be merged with their most suitable neighboring segment. | 10 |

**Appendix S4.** All classes OBIA-SVM.

| **Class** | **Reference data** | |  |  |  |  |  |  |  |  |  |  |
| --- | --- | --- | --- | --- | --- | --- | --- | --- | --- | --- | --- | --- |
|  | **Purple lupine** | **Pink lupine** | **Greenish lupine** | **Veg. shadow** | **Veg.** | **Road shadow** | **Road** | **Branches** | **Veg. dry** | **Precision (%)** | **Recall (%)** | **F1 score (%)** |
| **Purple lupine** | **6465** | 34 | 150 | 49 | 321 | 212 | 193 | 731 | 21 | 96.3 | 79.1 | 86.8 |
| **Pink lupine** | 65 | **528** | 15 | 4 | 14 | 12 | 220 | 185 | 160 | 91.7 | 43.9 | 59.4 |
| **Greenish lupine** | 137 | 2 | **580** | 20 | 358 | 2 | 110 | 202 | 389 | 76.4 | 32.2 | 45.3 |
| **Veg. shadow** | 0 | 0 | 0 | **899** | 100 | 0 | 0 | 1 | 0 | 83.0 | 89.9 | 86.3 |
| **Veg.** | 0 | 0 | 3 | 8 | **989** | 0 | 0 | 0 | 0 | 50.8 | 98.9 | 67.1 |
| **Road shadow** | 7 | 0 | 0 | 0 | 0 | **906** | 83 | 4 | 0 | 78.7 | 90.6 | 84.2 |
| **Road** | 0 | 3 | 0 | 0 | 0 | 1 | **116** | 880 | 0 | 15.1 | 11.6 | 13.1 |
| **Branches** | 38 | 9 | 7 | 21 | 62 | 18 | 38 | **371** | 36 | 15.6 | 61.8 | 24.9 |
| **Veg. dry** | 0 | 0 | 4 | 82 | 103 | 0 | 9 | 1 | **801** | 56.9 | 80.1 | 66.6 |
| **Total area of land cover (%)** | 37.2 | 0.1 | 0.5 | 15.1 | 25.6 | 2.7 | 3.3 | 10.9 | 4.6 |  |  |  |

**Appendix S5.** All classes OBIA-RF.

| **Class** | **Reference data** | |  |  |  |  |  |  |  |  |  |  |
| --- | --- | --- | --- | --- | --- | --- | --- | --- | --- | --- | --- | --- |
|  | **Purple lupine** | **Pink lupine** | **Greenish lupine** | **Veg. shadow** | **Veg.** | **Road shadow** | **Road** | **Branches** | **Veg. dry** | **Precision (%)** | **Recall (%)** | **F1 score (%)** |
| **Purple lupine** | **6716** | 66 | 7 | 44 | 81 | 213 | 556 | 401 | 92 | 93.0 | 82.1 | 87.2 |
| **Pink lupine** | 121 | **623** | 0 | 1 | 16 | 0 | 195 | 183 | 64 | 80.5 | 51.8 | 63.0 |
| **Greenish lupine** | 227 | 10 | **26** | 3 | 283 | 6 | 313 | 159 | 773 | 76.5 | 1.4 | 2.8 |
| **Veg. shadow** | 0 | 0 | 0 | **987** | 13 | 0 | 0 | 0 | 0 | 86.7 | 98.7 | 92.3 |
| **Veg.** | 0 | 0 | 0 | 13 | **983** | 0 | 0 | 0 | 4 | 61.6 | 98.3 | 75.7 |
| **Road shadow** | 5 | 0 | 0 | 0 | 0 | **962** | 32 | 1 | 0 | 78.7 | 96.2 | 86.5 |
| **Road** | 0 | 3 | 0 | 0 | 0 | 0 | **942** | 55 | 0 | 43.1 | 94.2 | 59.1 |
| **Branches** | 154 | 65 | 1 | 44 | 19 | 40 | 38 | **189** | 50 | 19.1 | 31.5 | 23.8 |
| **Veg. dry** | 0 | 7 | 0 | 46 | 202 | 2 | 112 | 2 | **629** | 39.0 | 62.9 | 48.2 |
| **Total area of land cover (%)** | 37.7 | 0.1 | 0 | 18.6 | 22.8 | 3.6 | 12.6 | 1.5 | 3.0 |  |  |  |

**Appendix S6.** All classes PBC-SVM.

| **Class** | **Reference data** | |  |  |  |  |  |  |  |  |  |  |
| --- | --- | --- | --- | --- | --- | --- | --- | --- | --- | --- | --- | --- |
|  | **Purple lupine** | **Pink lupine** | **Greenish lupine** | **Veg. shadow** | **Veg.** | **Road shadow** | **Road** | **Branches** | **Veg. dry** | **Precision (%)** | **Recall (%)** | **F1 score (%)** |
| **Purple lupine** | **6080** | 39 | 107 | 32 | 127 | 1264 | 38 | 489 | 0 | 93.3 | 74.4 | 82.8 |
| **Pink lupine** | 10 | **618** | 17 | 3 | 11 | 3 | 264 | 205 | 72 | 89.8 | 51.4 | 65.4 |
| **Greenish lupine** | 156 | 0 | **1182** | 9 | 156 | 3 | 37 | 179 | 78 | 85.3 | 65.7 | 74.2 |
| **Veg. shadow** | 0 | 0 | 0 | **971** | 22 | 0 | 0 | 5 | 2 | 86.3 | 97.1 | 91.4 |
| **Veg.** | 0 | 0 | 0 | 3 | **997** | 0 | 0 | 0 | 0 | 68.9 | 99.7 | 81.5 |
| **Road shadow** | 168 | 3 | 0 | 0 | 0 | **785** | 16 | 28 | 0 | 37.7 | 78.5 | 51.0 |
| **Road** | 0 | 14 | 1 | 0 | 0 | 0 | **962** | 23 | 0 | 70.7 | 96.2 | 81.5 |
| **Branches** | 103 | 9 | 70 | 11 | 29 | 26 | 44 | **284** | 24 | 23.4 | 47.3 | 31.3 |
| **Veg. dry** | 0 | 5 | 8 | 96 | 105 | 0 | 0 | 3 | **783** | 81.6 | 78.3 | 79.9 |
| **Total area of land cover (%)** | 1.5 | 0.6 | 1.3 | 26.6 | 36.6 | 3.8 | 18.8 | 2.4 | 8.4 |  |  |  |

**Appendix S7.** All classes PBC-RF.

| **Class** | **Reference data** | |  |  |  |  |  |  |  |  |  |  |
| --- | --- | --- | --- | --- | --- | --- | --- | --- | --- | --- | --- | --- |
|  | **Purple lupine** | **Pink lupine** | **Greenish lupine** | **Veg. shadow** | **Veg.** | **Road shadow** | **Road** | **Branches** | **Veg. dry** | **Precision (%)** | **Recall (%)** | **F1 score (%)** |
| **Purple lupine** | **6427** | 72 | 88 | 20 | 88 | 973 | 39 | 466 | 3 | 89.5 | 78.6 | 83.7 |
| **Pink lupine** | 28 | **646** | 16 | 1 | 7 | 5 | 225 | 205 | 70 | 79.8 | 53.7 | 64.2 |
| **Greenish lupine** | 204 | 2 | **943** | 3 | 193 | 3 | 45 | 209 | 198 | 84.3 | 52.4 | 64.6 |
| **Veg. shadow** | 0 | 0 | 0 | **984** | 13 | 0 | 0 | 3 | 0 | 85.6 | 98.4 | 91.5 |
| **Veg.** | 0 | 0 | 0 | 0 | **999** | 0 | 0 | 0 | 1 | 69.3 | 99.9 | 81.8 |
| **Road shadow** | 372 | 7 | 0 | 0 | 0 | **577** | 13 | 31 | 0 | 36.4 | 57.7 | 44.6 |
| **Road** | 9 | 63 | 0 | 0 | 0 | 1 | **902** | 25 | 0 | 71.1 | 90.2 | 79.5 |
| **Branches** | 139 | 15 | 63 | 20 | 23 | 27 | 45 | **243** | 25 | 20.5 | 40.5 | 27.2 |
| **Veg. dry** | 0 | 5 | 9 | 122 | 119 | 0 | 0 | 2 | **743** | 71.4 | 74.3 | 72.8 |
| **Total area of land cover (%)** | 3.1 | 1.5 | 1.0 | 26.6 | 39.2 | 2.6 | 17.5 | 2.3 | 6.2 |  |  |  |

**Appendix S8.** NoRC OBIA-SVM.

| **Class** | **Reference data** | |  |  |  |  |  |  |  |  |
| --- | --- | --- | --- | --- | --- | --- | --- | --- | --- | --- |
|  | **Purple lupine** | **Pink lupine** | **Greenish lupine** | **Veg. shadow** | **Veg.** | **Branches** | **Veg. dry** | **Precision (%)** | **Recall (%)** | **F1 score (%)** |
| **Purple lupine** | **6893** | 33 | 208 | 58 | 317 | 636 | 31 | 95.6 | 84.3 | 89.6 |
| **Pink lupine** | 107 | **748** | 18 | 5 | 14 | 142 | 169 | 91.3 | 62.2 | 74.0 |
| **Greenish lupine** | 145 | 6 | **683** | 8 | 356 | 157 | 445 | 73.7 | 37.9 | 50.1 |
| **Veg. shadow** | 0 | 0 | 0 | **899** | 101 | 0 | 0 | 85.6 | 89.9 | 87.7 |
| **Veg.** | 0 | 0 | 3 | 0 | **997** | 0 | 0 | 51.6 | 99.7 | 68.0 |
| **Branches** | 69 | 32 | 10 | 23 | 66 | **359** | 41 | 27.7 | 59.8 | 37.9 |
| **Veg. dry** | 0 | 0 | 5 | 57 | 81 | 1 | **856** | 55.5 | 85.6 | 67.3 |
| **Total area of land cover (%)** | 39.3 | 1.3 | 0.7 | 12.8 | 28.1 | 13.1 | 4.8 |  |  |  |

**Appendix S9.** NoRC OBIA-RF.

| **Class** | **Reference data** | |  |  |  |  |  |  |  |  |
| --- | --- | --- | --- | --- | --- | --- | --- | --- | --- | --- |
|  | **Purple lupine** | **Pink lupine** | **Greenish lupine** | **Veg. shadow** | **Veg.** | **Branches** | **Veg. dry** | **Precision (%)** | **Recall (%)** | **F1 score (%)** |
| **Purple lupine** | **6699** | 135 | 319 | 70 | 93 | 602 | 258 | 94.6 | 81.9 | 87.8 |
| **Pink lupine** | 107 | **807** | 23 | 0 | 27 | 89 | 150 | 76.0 | 67.1 | 71.3 |
| **Greenish lupine** | 146 | 42 | **326** | 29 | 294 | 157 | 806 | 43.8 | 18.1 | 25.6 |
| **Veg. shadow** | 1 | 0 | 0 | **970** | 29 | 0 | 0 | 81.6 | 97.0 | 88.7 |
| **Veg.** | 0 | 0 | 4 | 9 | **978** | 0 | 9 | 61.6 | 97.8 | 75.6 |
| **Branches** | 132 | 74 | 12 | 46 | 31 | **220** | 85 | 20.5 | 36.7 | 26.3 |
| **Veg. dry** | 0 | 4 | 60 | 64 | 136 | 3 | **733** | 35.9 | 73.3 | 48.2 |
| **Total area of land cover (%)** | 40.0 | 1.3 | 0.8 | 18.7 | 23.0 | 11.5 | 4.7 |  |  |  |

**Appendix S10.** NoRC PBC-SVM.

| **Class** | **Reference data** | |  |  |  |  |  |  |  |  |
| --- | --- | --- | --- | --- | --- | --- | --- | --- | --- | --- |
|  | **Purple lupine** | **Pink lupine** | **Greenish lupine** | **Veg. shadow** | **Veg.** | **Branches** | **Veg. dry** | **Precision (%)** | **Recall (%)** | **F1 score (%)** |
| **Purple lupine** | **7420** | 26 | 133 | 54 | 108 | 432 | 3 | 95.4 | 90.8 | 93.0 |
| **Pink lupine** | 29 | **794** | 17 | 5 | 20 | 242 | 96 | 94.0 | 66.0 | 77.5 |
| **Greenish lupine** | 190 | 5 | **1106** | 14 | 156 | 199 | 130 | 82.9 | 61.4 | 70.6 |
| **Veg. shadow** | 0 | 0 | 0 | **952** | 13 | 5 | 30 | 82.8 | 95.2 | 88.6 |
| **Veg.** | 0 | 0 | 0 | 3 | **994** | 0 | 3 | 70.8 | 99.4 | 82.7 |
| **Branches** | 136 | 14 | 70 | 14 | 24 | **313** | 29 | 26.2 | 52.2 | 34.9 |
| **Veg. dry** | 0 | 6 | 8 | 108 | 88 | 2 | **788** | 73.0 | 78.8 | 75.8 |
| **Total area of land cover (%)** | 6.3 | 11.9 | 1.3 | 25.6 | 34.7 | 8.8 | 11.4 |  |  |  |

**Appendix S11.** NoRC PBC-RF.

| **Class** | **Reference data** | |  |  |  |  |  |  |  |  |
| --- | --- | --- | --- | --- | --- | --- | --- | --- | --- | --- |
|  | **Purple lupine** | **Pink lupine** | **Greenish lupine** | **Veg. shadow** | **Veg.** | **Branches** | **Veg. dry** | **Precision (%)** | **Recall (%)** | **F1 score (%)** |
| **Purple lupine** | **7322** | 59 | 102 | 13 | 101 | 577 | 2 | 94.5 | 89.6 | 91.9 |
| **Pink lupine** | 59 | **803** | 11 | 1 | 9 | 232 | 88 | 89.6 | 66.7 | 76.5 |
| **Greenish lupine** | 205 | 3 | **960** | 3 | 212 | 238 | 179 | 83.8 | 53.3 | 65.2 |
| **Veg. shadow** | 0 | 0 | 0 | **977** | 18 | 4 | 1 | 86.7 | 97.7 | 91.9 |
| **Veg.** | 0 | 0 | 0 | 0 | **1000** | 0 | 0 | 65.7 | 100.0 | 79.3 |
| **Branches** | 165 | 23 | 64 | 17 | 33 | **270** | 28 | 20.4 | 45.0 | 28.1 |
| **Veg. dry** | 0 | 8 | 9 | 116 | 148 | 2 | **717** | 70.6 | 71.7 | 71.2 |
| **Total area of land cover (%)** | 6.5 | 11.9 | 1.0 | 27.0 | 40.1 | 8.5 | 5.1 |  |  |  |

**Appendix S12.** NoRBC OBIA-SVM.

| **Class** | **Reference data** | |  |  |  |  |  |  |  |
| --- | --- | --- | --- | --- | --- | --- | --- | --- | --- |
|  | **Purple lupine** | **Pink lupine** | **Greenish lupine** | **Veg. shadow** | **Veg.** | **Veg. dry** | **Precision (%)** | **Recall (%)** | **F1 score (%)** |
| **Purple lupine** | **7354** | 49 | 244 | 115 | 373 | 41 | 95.8 | 89.9 | 92.8 |
| **Pink lupine** | 132 | **782** | 15 | 7 | 23 | 244 | 92.9 | 65.0 | 76.5 |
| **Greenish lupine** | 191 | 11 | **648** | 17 | 383 | 550 | 70.9 | 36.0 | 47.8 |
| **Veg. shadow** | 0 | 0 | 0 | **907** | 93 | 0 | 80.6 | 90.7 | 85.4 |
| **Veg.** | 0 | 0 | 2 | 2 | **996** | 0 | 50.8 | 99.6 | 67.3 |
| **Veg. dry** | 0 | 0 | 5 | 77 | 94 | **824** | 49.7 | 82.4 | 62.0 |
| **Total area of land cover (%)** | 41.4 | 11.6 | 0.7 | 13.7 | 27.8 | 4.8 |  |  |  |

**Appendix S13.** NoRBC OBIA-RF.

| **Class** | **Reference data** | |  |  |  |  |  |  |  |
| --- | --- | --- | --- | --- | --- | --- | --- | --- | --- |
|  | **Purple lupine** | **Pink lupine** | **Greenish lupine** | **Veg. shadow** | **Veg.** | **Veg. dry** | **Precision (%)** | **Recall (%)** | **F1 score (%)** |
| **Purple lupine** | **7436** | 169 | 225 | 73 | 81 | 192 | 94.7 | 90.9 | 92.8 |
| **Pink lupine** | 124 | **842** | 41 | 0 | 36 | 160 | 78.8 | 70.0 | 74.2 |
| **Greenish lupine** | 296 | 52 | **408** | 18 | 362 | 664 | 58.7 | 22.7 | 32.7 |
| **Veg. shadow** | 0 | 0 | 0 | **975** | 25 | 0 | 88.0 | 97.5 | 92.5 |
| **Veg.** | 0 | 0 | 1 | 5 | **989** | 5 | 60.7 | 98.9 | 75.2 |
| **Veg. dry** | 0 | 5 | 20 | 37 | 137 | **801** | 44.0 | 80.1 | 56.8 |
| **Total area of land cover (%)** | 41.1 | 11.5 | 0.5 | 18.0 | 24.7 | 4.2 |  |  |  |

**Appendix S14.** NoRBC PBC-SVM.

| **Class** | **Reference data** | |  |  |  |  |  |  |  |
| --- | --- | --- | --- | --- | --- | --- | --- | --- | --- |
|  | **Purple lupine** | **Pink lupine** | **Greenish lupine** | **Veg. shadow** | **Veg.** | **Veg. dry** | **Precision (%)** | **Recall (%)** | **F1 score (%)** |
| **Purple lupine** | **7668** | 54 | 223 | 118 | 91 | 22 | 96.1 | 93.8 | 94.9 |
| **Pink lupine** | 54 | **955** | 23 | 9 | 21 | 141 | 91.7 | 79.4 | 85.1 |
| **Greenish lupine** | 256 | 22 | **1234** | 15 | 160 | 113 | 82.9 | 68.6 | 75.1 |
| **Veg. shadow** | 2 | 0 | 0 | **936** | 57 | 5 | 77.4 | 93.6 | 84.7 |
| **Veg.** | 0 | 0 | 0 | 6 | **992** | 2 | 69.0 | 99.2 | 81.4 |
| **Veg. dry** | 1 | 10 | 8 | 126 | 117 | **738** | 72.3 | 73.8 | 73.0 |
| **Total area of land cover (%)** | 7.9 | 18.0 | 1.4 | 23.7 | 39.0 | 9.8 |  |  |  |

**Appendix S15.** NoRBC PBC-RF.

| **Class** | **Reference data** | |  |  |  |  |  |  |  |
| --- | --- | --- | --- | --- | --- | --- | --- | --- | --- |
|  | **Purple lupine** | **Pink lupine** | **Greenish lupine** | **Veg. shadow** | **Veg.** | **Veg. dry** | **Precision (%)** | **Recall (%)** | **F1 score (%)** |
| **Purple lupine** | **7882** | 57 | 117 | 21 | 95 | 4 | 95.2 | 96.4 | 95.8 |
| **Pink lupine** | 101 | **963** | 28 | 1 | 8 | 102 | 91.2 | 80.0 | 85.3 |
| **Greenish lupine** | 300 | 32 | **1062** | 6 | 239 | 161 | 87.2 | 59.0 | 70.4 |
| **Veg. shadow** | 0 | 0 | 0 | **981** | 19 | 0 | 86.4 | 98.1 | 91.9 |
| **Veg.** | 0 | 0 | 0 | 0 | **999** | 1 | 65.8 | 99.9 | 79.3 |
| **Veg. dry** | 0 | 4 | 11 | 126 | 159 | **700** | 72.3 | 70.0 | 71.1 |
| **Total area of land cover (%)** | 8.8 | 17.4 | 1.2 | 25.1 | 41.9 | 5.5 |  |  |  |

**Appendix S16.** Classified raster maps before (left) and after (right) masking of roads. The left image shows the initial classification, where the road is misclassified as pink and purple lupine. The right image illustrates the result after masking and excluding the road surface, reducing misclassification in the study area.

**Appendix S17.** Over- and underestimation of lupine occurrence. The red areas represent underestimation, while the yellow areas indicate overestimation in relation to the validation data. The points represent validation data for lupine, and the blue polygons show the classified lupine occurrence.
